# A global research agenda on public health and social measures during emergencies

**DOI:** 10.2471/BLT.23.289959

**Published:** 2023-09-28

**Authors:** Ramona Ludolph, Ryoko Takahashi, Zubin Cyrus Shroff, Monika Kosinska, Tanja Schmidt, Huda Haidar Anan, Fatima Arifi, Abdoulaye Yam, Kumanan Rasanathan, Abraham Aseffa, Phuong Nam Nguyen, Masaya Kato, Aarti Garg, Tshewang Dorji, Andrea Villalobos, Victoria Haldane, Tim Nguyen, Sylvie Briand

**Affiliations:** a Epidemic and Pandemic Preparedness and Prevention Department, WHO Health Emergencies Programme, World Health Organization, 20 Avenue Appia, 1211 Geneva, Switzerland.; b Alliance for Health Policy and Systems Research, World Health Organization, Geneva, Switzerland.; c Department of Social Determinants of Health, World Health Organization, Geneva, Switzerland.; d WHO Health Emergencies Programme, World Health Organization Regional Office for Europe, Copenhagen, Denmark.; e WHO Health Emergencies Programme, World Health Organization Regional Office for the Eastern Mediterranean, Cairo, Egypt.; f Emergency Preparedness and Response Cluster, World Health Organization Regional Office for Africa, Brazzaville, Congo.; g The Special Programme for Research and Training in Tropical Diseases, World Health Organization, Geneva, Switzerland.; h WHO Health Emergencies Programme, World Health Organization Regional Office for the Western Pacific, Manila, Philippines.; i WHO Health Emergencies Programme, World Health Organization Regional Office for South-East Asia, New Delhi, India.; j Public Health Emergencies, Pan American Health Organization, Washington, DC, United States of America.

## Abstract

The importance of strong coordination for research on public health and social measures was highlighted at the Seventy-fourth World Health Assembly in 2021. This article describes efforts undertaken by the World Health Organization (WHO) to develop a global research agenda on the use of public health and social measures during health emergencies. This work includes a multistep process that started with a global technical consultation convened by WHO in September 2021. The consultation included experts from around the world and from a wide range of disciplines, such as public health, education, tourism, finance and social sciences, and aimed to identify research and implementation approaches based on lessons learnt during the coronavirus disease 2019 pandemic. To prepare for future epidemics and pandemics, it is essential to adopt a more robust, comparable and systematic research approach to public health and social measures. Such comprehensive approach will better inform agile, balanced and context-specific implementation decisions during future emergencies. This article describes the methods used to develop global research priorities for public health and social measures and the next steps needed.

## Résumé

La soixante-quatorzième Assemblée mondiale de la Santé en 2021 a souligné l'importance d'une coordination solide pour la recherche sur la santé publique et les mesures sociales. Le présent article décrit les efforts entrepris par l'Organisation mondiale de la santé (OMS) pour élaborer un programme de recherche mondial sur l'utilisation des mesures de santé publique et des mesures sociales lors de situations d'urgence sanitaire. Ce travail comprend un processus en plusieurs étapes qui a commencé par une consultation technique mondiale organisée par l'OMS en septembre 2021. La consultation a réuni des experts du monde entier issus d'un large éventail de disciplines telles que la santé publique, l'éducation, le tourisme, la finance et les sciences sociales. Elle visait à identifier des approches de recherche et de mise en œuvre fondées sur les enseignements tirés de la pandémie de maladie à coronavirus de 2019. Pour se préparer aux futures épidémies et pandémies, il est essentiel d'adopter une approche de recherche plus solide, comparable et systématique en matière de santé publique et de mesures sociales. Cette approche globale permettra de mieux éclairer les décisions de mise en œuvre agiles, équilibrées et adaptées au contexte lors des futures situations d'urgence. Le présent article décrit les méthodes appliquées pour définir les priorités mondiales de recherche en matière de santé publique et de mesures sociales, ainsi que les prochaines étapes à franchir.

## Resumen

En la 74.ª Asamblea Mundial de la Salud, celebrada en 2021, se destacó la importancia de una sólida coordinación en la investigación sobre salud pública y medidas sociales. Este artículo describe los esfuerzos que ha emprendido la Organización Mundial de la Salud (OMS) para desarrollar un programa mundial de investigación sobre el uso de medidas sociales y de salud pública durante las emergencias sanitarias. Este trabajo incluye un proceso de varios pasos que comenzó con una consulta técnica mundial que convocó la OMS en septiembre de 2021. La consulta incluyó a expertos de todo el mundo y de una gran variedad de disciplinas, como la salud pública, la educación, el turismo, las finanzas y las ciencias sociales, y tuvo como objetivo identificar enfoques de investigación y aplicación basados en las lecciones aprendidas durante la pandemia de la enfermedad por coronavirus de 2019. Para prepararse ante futuras epidemias y pandemias, es esencial adoptar un enfoque de investigación más sólido, comparable y sistemático en materia de salud pública y medidas sociales. Este enfoque integral informará mejor las decisiones de aplicación ágiles, equilibradas y adaptadas al contexto durante futuras emergencias. En este artículo se describen los métodos utilizados para elaborar las prioridades mundiales de investigación sobre salud pública y medidas sociales, así como los próximos pasos necesarios.

## ملخص

تم تسليط الضوء على أهمية التنسيق الكامل للأبحاث في الصحة العامة والتدابير الاجتماعية، في جمعية الصحة العالمية الرابعة والسبعين في عام 2021. تصف هذه المقالة الجهود التي بذلتها منظمة الصحة العالمية (WHO) لوضع أجندة للأبحاث العالمية حول اتباع تدابير الصحة العامة والتدابير الاجتماعية أثناء حالات الطوارئ الصحية. ويتضمن هذا العمل عملية متعددة الخطوات، بدأت باستشارات فنية عالمية عقدتها منظمة الصحة العالمية في سبتمبر/أيلول 2021. وشملت الاستشارات خبراء من جميع أنحاء العالم، ومن مجموعة واسعة من التخصصات، مثل الصحة العامة، والتعليم، والسياحة، والمالية، والعلوم الاجتماعية. وتهدف الاستشارات إلى تحديد أساليب البحث والتنفيذ بناءً على الدروس المستفادة خلال جائحة مرض فيروس كورونا 2019. للاستعداد للأوبئة والجوائح المستقبلية، من الضروري انتهاج أسلوب بحثي أكثر فعالية، وقابلية للمقارنة، وتنظيمًا فيما يتعلق بالصحة العامة والتدابير الاجتماعية. ومثل هذا الأسلوب الشامل سيؤدي اتخاذ قرارات التنفيذ المرنة والمتوازنة ومحددة السياق، خلال حالات الطوارئ المستقبلية، بشكل أفضل. توضح هذه المقالة الطرق المستخدمة لتحديد أولويات البحث العالمية للصحة العامة، والتدابير الاجتماعية، والخطوات التالية اللازمة.

## 摘要

2021 年第七十四届世界卫生大会强调了加强协调公共卫生和社会措施研究的重要性。本文介绍了世界卫生组织 (WHO) 为制定关于在突发卫生事件期间使用的公共卫生和社会措施的全球研究议程所做的努力。这项工作包括一个多阶段的进程，该进程始于世界卫生组织于 2021 年 9 月召开的全球技术磋商会。此次磋商会包括来自世界各地和公共卫生、教育、旅游、金融和社会科学等各个学科的专家，旨在根据新型冠状病毒病疫情期间吸取的经验教训，确定研究和实施方法。为了为未来的流行病和疫情做好准备，我们必须对公共卫生和社会措施采取更有力、更具可比性和更系统的研究方法。这种全面性的方法将在未来突发事件期间为制定灵活、平衡和针对具体情况的实施决策更好地提供信息。本文介绍了用于制定公共卫生和社会措施的全球研究优先事项的方法以及接下来需要采取的步骤。

## Резюме

На семьдесят четвертой сессии Всемирной ассамблеи здравоохранения в 2021 году подчеркивалась важность эффективной координации исследований в области общественного здоровья и социальной защиты. В данной статье описываются усилия, предпринимаемые Всемирной организацией здравоохранения (ВОЗ) для разработки глобальной программы научных исследований по использованию мер в области общественного здравоохранения и социальной защиты во время чрезвычайных ситуаций в области здравоохранения. Эта работа предусматривает многоэтапный процесс, который начался с глобальной технической консультации, созванной ВОЗ в сентябре 2021 года. В консультациях приняли участие эксперты из разных стран мира, представляющие широкий спектр дисциплин, таких как общественное здравоохранение, образование, туризм, финансы и социальные науки, и ставившие перед собой задачу определить подходы к исследованиям и внедрению, основанные на опыте, накопленном во время пандемии коронавирусной болезни 2019 года. Для подготовки к предстоящим эпидемиям и пандемиям необходимо использовать более надежный, сопоставимый и систематический исследовательский подход к мерам в области общественного здравоохранения и социальной защиты. Использование такого комплексного подхода позволит более эффективно принимать оперативные, взвешенные и учитывающие конкретные условия решения по реализации программ в чрезвычайных ситуациях в будущем. В данной статье описываются используемые методы разработки приоритетов глобальных исследований в области общественного здравоохранения и социальной защиты, а также необходимые дальнейшие шаги.

## Introduction

Public health and social measures refer to non-pharmaceutical interventions implemented by individuals, communities and governments at all levels. Public health and social measures are a key strategy to offer increased individual and community protection during infectious disease outbreaks, alongside medical countermeasures such as vaccines and therapeutics.^1^^,^^2^ While the suitability and effectiveness of each intervention is dependent on a pathogen’s mode of transmission, public health and social measures can in general reduce the risk and scale of transmission by reducing the number of transmission-relevant exposure situations and/or making them safer. By limiting or eliminating transmission, public health and social measures reduce the prevalence of severe disease and death and thus lower pressure on the health-care system. The reduced pressure supports the continuity of essential health services and buys time to develop and implement vaccines and therapeutics.

During the coronavirus disease 2019 (COVID-19) pandemic, public health and social measures were applied globally on an exceptional scale and for an unprecedented length of time. Interventions applied during the COVID-19 pandemic, for example, ranged from personal protection measures, such as mask-wearing and handwashing, to social measures including physical distancing and modified mass gatherings and schooling, to international travel and trade measures.^3^ Public health and social measures have protected both lives and livelihoods,^4^ but social and international travel and trade measures were often highly disruptive to societal life. These measures were implemented over prolonged periods of time without adequate evidence of their effectiveness, and led to negative health, social and economic consequences for individuals and societies. These repercussions included increased economic hardship due to job losses, disrupted learning and development, interruptions to routine public health programmes, worsened mental health, decreased physical activity, and increased gender and social inequities.^5^^–^^7^ Vulnerable populations and marginalized communities were particularly affected by the negative consequences.^8^ To protect the health of communities, policies and decisions about public health and social measures need to be informed by the best available evidence to weigh their health benefits against potentially negative consequences.

Developing robust methods and innovative study designs to measure the effectiveness of public health and social measures, and their health, social and economic impacts, as well as the factors influencing adherence to measures, is crucial to supporting emergency preparedness and response activities.^9^ However, the systematic evaluation of public health and social measures has proven difficult due to the complex nature of the interventions and ethical, legal, resource and feasibility limitations of conducting research, especially (quasi-) randomized (controlled) trials, in an emergency context.

Given these challenges, the World Health Organization (WHO) is leading the development of a global research agenda aimed at gaining a better understanding of the effectiveness and impact of public health and social measures during health emergencies over the next 10 years. The research agenda is part of a larger initiative, launched in June 2021, that aims to strengthen the global evidence base to provide actionable and evidence-informed guidance on public health and social measures.^10^ In this paper, we describe the method underlying the development of the global public health and social measures research agenda and the results of the first stage of the process.

## Rationale for the agenda

The importance of strong coordination for research and policy related to public health and social measures was emphasized during the Seventy-fourth World Health Assembly in 2021. At this meeting, Member States requested the Director-General to “develop a global framework to generate, monitor, compare and evaluate research and policies on public health and social interventions and assess their broader impact, in order to harness global knowledge and expertise and to translate evidence into effective health emergency and preparedness policies.”^11^

Using the breadth of global knowledge and expertise on public health and social measures is crucial to enhancing evidence-informed decision-making about the implementation, combination, adjustment and communication of these measures before, during and after emergencies. Indeed, understanding the effectiveness and broader impacts of public health and social measures forms a critical basis for more systematic and equitable decision-making about their implementation, including continual adjustment during different phases of a health emergency. However, especially in the earlier stages of the COVID-19 pandemic in 2020–2021 when medical countermeasures were not available or equitably distributed, various combinations of public health and social measures were implemented. So-called lockdowns were the most stringent interventions, as all aspects of people’s lives were affected and personal freedoms restricted. Ethical considerations and the inherently complex, multidisciplinary and multisectoral nature of public health and social measures posed challenges not only to conducting research about their effectiveness and impact, but also to making decisions based on this research.^12^ From primary data collection to timely and well-communicated dissemination of results relevant to decision-makers, the pandemic highlighted the current limitations in how public health and social measures research is prioritized, conducted, coordinated and used for policy-making during health emergencies. Thus, a global research agenda is necessary to guide a cohesive and systematic approach to developing robust evidence about public health and social measures.

## Methodological approach

The research agenda is being developed according to WHO’s guidance on undertaking priority-setting exercises for research.^13^ This process has multiple stages and includes public surveys and expert consultations (Box 1). The iterative process ensures that the research agenda will be relevant to the ongoing COVID-19 pandemic, and will also allow the integration of research priorities for future health emergencies caused by other respiratory pathogens. The objectives of the multistep method are to: (i) ensure that urgent research priorities are established to guide the ongoing and emerging research during the COVID-19 pandemic (stages I and II); (ii) allow for finalization of global research priorities relevant to COVID-19 based on the results of a global evidence review; (iii) expand the research priorities to pathogens other than COVID-19 to ensure their longer-term relevance, and contribute to emergency preparedness efforts (stage III); and (iv) include a wide range of stakeholders and sectors in the priority-setting exercise to reflect global diversity.

Box 1 **Multistage development process of the WHO global research agenda on public health and social measures, 2021–2030**

***Stage I***

- Developed a background paper outlining six research themes (August 2021).
- Discussed research themes and developed a draft research agenda at the WHO global technical consultation on public health and social measures during health emergencies (August–September 2021).

***Stage II***

- Conducted a public survey to validate the research themes and identify priority research questions for public health and social measures related to the COVID-19 pandemic (August–October 2022).

***Stage III***

- Conducted a public survey to identify priority research questions for public health and social measures in relation to COVID-19 and other pathogens (September 2023).

***Stage IV***

- Discuss the results of the surveys at the second WHO global consultation on public health and social measures during health emergencies (November 2023).
- Publish the global research agenda on public health and social measures (February 2024).

COVID-19: coronavirus disease 2019; WHO: World Health Organization.

### Stage I: developing a research agenda

From 31 August to 2 September 2021, WHO convened a global technical consultation on using public health and social measures during health emergencies, to identify research and implementation approaches for the future use of these measures based on lessons learnt during the COVID-19 pandemic.^14^ The virtual consultation brought together more than 100 experts from around the world and from a wide range of disciplines, such as public health, education, tourism, finance and social sciences, to discuss multiple topics, including the global public health and social measures research agenda. WHO, together with researchers from the Behavioural, Environmental, Social and Systems Interventions collaboration,^15^ prepared a background paper for discussion at the consultation.^13^ Participants validated and refined six research themes as overarching categories for the future exercise in setting research priorities (Box 2).

Box 2 **Themes to guide a global research agenda on public health and social measures defined at the WHO global technical consultation, 2021**

***1. Mapping existing research to identify knowledge gaps***

- Previous outbreaks and the coronavirus disease 2019 (COVID-19) pandemic have generated a substantial body of evidence on public health and social measures that can be used to inform policy and identify research gaps.

***2. Measuring the effectiveness and impact of public health and social measures on transmission, morbidity and mortality***

- Due to simultaneous implementation of multiple public health and social measures and a lack of mechanisms to prospectively collect data, large knowledge gaps still exist on the usefulness of single and combined measures and their effect on virus transmission and their direct effect on morbidity and mortality.

***3. Assessing the impact of public health and social measures on health, social and economic outcomes***

- As complex interventions, public health and social measures have significant, indirect consequences on health, and social and economic outcomes at individual and societal levels.
- As the social and economic impacts are influenced by the determinants of health, any negative consequences in these areas will ultimately affect the health and well-being of individuals and societies.

***4. Building preparedness for future health emergencies: resilience and response capacity***

- As public health and social measures are a key strategy to limit transmission, especially in the absence of pharmaceutical interventions, they will continue to play an important role in ensuring preparedness for health emergencies.
- It is possible to prepare evidence-informed and equitable public health and social measures and plan for their balanced implementation in future, both to increase response capacity and build resilience against the potential negative consequences of an intervention.

***5. Promoting uptake of and adherence to public health and social measures***

- The effectiveness and impact of public health and social measures largely depend on whether the public engages in the protective measures and adheres to them for as long as they are recommended.
- Pandemic fatigue, a lack of trust in authorities and science, and conflicting beliefs, values and preferences can lead to decreased uptake of and adherence to public health and social measures.

***6. Conducting methodological research to advance implementation and evaluation of public health and social measures***

- Evaluating the effectiveness of public health and social measures poses methodological challenges to the multidisciplinary research community. These challenges can be addressed by promoting meta-research on research coordination, infrastructure and preparedness, and methods of evidence synthesis and evaluation research. The range of research approaches to study public health and social measures needs to be expanded, including health policy and systems research and implementation research.

WHO: World Health Organization.Source: WHO, 2021.^14^

During the consultation, experts emphasized that research on public health and social measures needed to address immediate priority questions on the COVID-19 pandemic; reflect on lessons learnt from this and other pandemics; and also strengthen public health and social measures research methods, measures and implementation during non-emergency phases that focus on preparedness. Participants underscored that ensuring that research on public health and social measures considers multiple perspectives requires investing in and nurturing multidisciplinary research capacity. This aspect is particularly important in low- and middle-income countries and resource-constrained settings, where limited evidence exists on the implementation and effectiveness of, and adherence to, public health and social measures. Multidisciplinary teams – for example, consisting of epidemiologists, public health professionals, and social, behavioural and political scientists – are well-placed to adopt research models, such as implementation research and health policy and systems research, and to determine whether contextually relevant questions require traditional evaluation methods or more flexible study designs. Overall, participants highlighted the need for equitable and sustainable collaboration and distribution of funding across all countries. The global collaboration to strengthen evidence on public health and social measures also includes promoting the production of community-led research and its uptake at local and regional levels, especially in low- and middle-income countries. As such, the global public health and social measures research agenda must include funders to ensure there is alignment between funding and research priorities.

Experts at the consultation recommended five key actions to develop the global research agenda on public health and social measures: (i) apply a multistep consultation approach; (ii) base the priority-setting exercise for long-term research on a global evidence review and gap analysis; (iii) include funders in the priority-setting exercise to ensure alignment between funding and research priorities; (iv) highlight the importance of implementation science, health policy, and systems and operational research; and (v) organize a series of public consultations, starting with an online public survey.

### Stage II: public consultation 

Following the drafting of a research agenda at the global expert consultation, an online survey was developed to allow for broader public involvement. This approach promoted inclusion of a wide range of global experts in the priority-setting exercise. The survey also served as the second stage of verification to improve the appropriateness and relevance of the suggested research themes discussed at the expert consultation. Along with a standard introduction and a section collecting demographic information, the survey asked participants to rank what they believed to be the top research priorities for public health and social measures in the immediate term (that is, the next 6–12 months) in relation to COVID-19. For each section of the survey, participants were asked to select up to three priority areas and then rank them from one to three.

The online survey was created using DataForm, a WHO online survey tool. The survey was first piloted with a small number of participants and subsequently revised based on their feedback. The survey invitation was published on the WHO public health and social measures website and disseminated through targeted outreach to: (i) the external public health and social measures working group under the Strategic and Technical Advisory Group on Infectious Hazards with Pandemic and Epidemic Potential, which is a network of experts who have collaborated with the public health and social measures secretariat; (ii) the internal WHO public health and social measures steering group; (iii) the WHO Research and Development Blueprint; (iv) the Evidence Collaborative on COVID-19 Network; (v) the Global Research Collaboration for Infectious Disease Preparedness; and (vi) social media, including Twitter and LinkedIn. The survey was in English and was open until 15 October 2022. A total of 202 respondents completed the survey.

The WHO public health and social measures secretariat synthesized the survey responses and conducted a preliminary analysis. The findings were subsequently reviewed by a group of senior research methodologists and are available on the website of the WHO public health and social measures initiative.^10^^,^^16^

### Stage III: identifying research priorities

Stage III of the priority-setting exercise for research will again consist of a public survey, and focus on strengthening the global public health and social measures evidence base in the medium- to long-term using a multihazards approach. The results of the global evidence review on public health and social measures will inform this phase, which consists of: (i) an appraisal of systematic reviews on the effectiveness and impact of public health and social measures during COVID-19; (ii) a scoping review of social protection measures to reduce the unintended negative consequences of public health and social measures; and (iii) a review of a set of case studies analysing country-level challenges to and facilitators of implementing public health and social measures, gathered during a consultation with public health stakeholders from 21 countries during a WHO meeting in June 2023.^14^

The questionnaire used for stage III of the priority-setting exercise will include questions on respondents’ professional background and demographics, and ask them to rank priority topics, study designs and products matching the themes presented in Box 2. The survey will be available for public participation on the WHO public health and social measures website from September 2023 onwards.

Survey distribution and the analysis of research priorities will be similar to the process described for stage II.

### Stage IV: finalizing the agenda 

The proposed medium- to long-term research agenda from stage III will be discussed with global experts at the second WHO global technical consultation on public health and social measures, planned for November 2023. To ensure continual awareness, uptake and effective implementation of the research priorities, the outcomes of this exercise will be widely disseminated through peer-reviewed articles, a summary report, newsletters and a public webinar. A consultation with research funders and philanthropic organizations is intended to identify funding opportunities to implement the research agenda. The progress of the global public health and social measures research agenda will be mapped through a WHO digital solution which will be available on the public health and social measures website from mid-2024 onwards.

## Conclusion

A multipronged approach, such as the one we describe here, is required to develop a comprehensive research agenda that addresses the challenges in assessing public health and social measures. As our preliminary work emphasized, such an approach must prioritize multidisciplinary perspectives, a range of study designs and effective knowledge translation. The COVID-19 pandemic and other past outbreak events have clearly demonstrated the value of perspectives beyond the traditional biomedical focus of pandemic responses.^17^ A strong focus on equity and the social determinants of health will ensure that the individual and societal consequences of implementation of public health and social measures are explored through community-focused research.^16^

While the global public health and social measures research agenda aims to promote multidisciplinary insight into the effectiveness of, negative consequences of, barriers to and enablers of adherence to public health and social measures, it is essential to increase the use of research evidence in decision-making. Guidance and support must be provided to decision-makers to consider context-specific implementation and precautionary principles, especially when facing unknowns and uncertainties in future health emergencies. Furthermore, it is imperative to identify an acceptable balance between the health benefits of public health and social measures, in terms of transmission reduction, and their drawbacks.

Past and current outbreaks have underscored that, among the many factors that shape decision-making, the absence of a comprehensive research agenda to bring together different disciplines risks decision-makers adopting a fragmented and incomplete approach, thereby undermining preparedness and response efforts. Through a collaborative approach, this research agenda can guide and catalyse research that is comparable, high quality, equity-focused and policy-relevant. Its primary aim is to support decision-makers from all relevant sectors to make evidence-informed, strategic decisions about public health and social measures, with the ultimate goal of safeguarding the health of communities during future health emergencies.
